# The Evidence for Allogeneic Hematopoietic Stem Cell Transplantation for Congenital Neutrophil Disorders: A Comprehensive Review by the Inborn Errors Working Party Group of the EBMT

**DOI:** 10.3389/fped.2019.00436

**Published:** 2019-10-24

**Authors:** Shahrzad Bakhtiar, Bella Shadur, Polina Stepensky

**Affiliations:** ^1^Division for Pediatric Stem Cell Transplantation and Immunology, University Hospital Frankfurt, Frankfurt, Germany; ^2^Department of Bone Marrow Transplantation and Cancer Immunotherapy, Hadassah Medical Center, Jerusalem, Israel; ^3^Department of Immunology, Garvan Institute of Medical Research, Darlinghurst, NSW, Australia; ^4^Graduate Research School, University of New South Wales, Kensington, NSW, Australia

**Keywords:** neutrophils, neutropenia, leukemia, granulocyte colony-stimulating factor, hematopoietic stem cell transplantation

## Abstract

Congenital disorders of the immune system affecting maturation and/or function of phagocytic leucocytes can result in severe infectious and inflammatory complications with high mortality and morbidity. Further complications include progression to MDS/AML in some cases. Allogeneic stem cell transplantation is the only curative treatment for most patients with these diseases. In this review, we provide a detailed update on indications and outcomes of alloHSCT for congenital neutrophil disorders, based on data from the available literature.

## Introduction

Congenital neutrophil disorders as a category of primary immunodeficiency (PID) can be classified in many ways, but a key point of distinction is whether the disorder is *quantitative*, or *qualitative* (1). The 2017 International Union of Immunological Societies (IUIS) Phenotypic Classification for Primary Immunodeficiencies divides neutrophil disorders into four broad categories: congenital neutropenia associated with or without syndromic disease, and functional neutrophil defects with or without syndromic disease (2). Affected patients can present with variable symptoms including recurrent infections, failure to thrive, and overwhelming septic episodes leading to high morbidity and mortality. Early and severe respiratory infections (e.g., *Burkholderia cepacia, Aspergillus* spp.), visceral abscesses, cellulitis, lymphadenitis, and granulomatous lesions are observed in patients suffering from CGD (3, 4). Some patients develop severe autoinflammatory complications underlining the role of neutrophils in autoinflammatory processes beyond microbial defense (5, 6). In many of these diseases there is a recognized risk of progression to myelodysplastic syndrome (MDS) and acute myeloid leukemia (AML) (1, 2, 7, 8). Treatment for neutrophil disorders classically comprises anti-microbial therapy, granulocyte-colony stimulating factor (G-CSF), and allogeneic hematopoietic stem cell transplantation (alloHSCT) (8).

In this review we provide an update on the evidence, indications and modalities of alloHSCT for the various congenital neutrophil disorders based on data from the available literature, excluding CGD which will be discussed elsewhere in this special edition. Also beyond the scope of this manuscript is neutropenia that features in predominantly lymphocyte immune deficiencies (e.g., some forms of severe combined immune deficiency, CD40L deficiency, etc.).

## Materials and Methods

We used the 2017 IUIS Phenotypic Classification for Primary Immunodeficiencies as the basis for this review (2). Data were gathered via an English-language Pubmed literature search whereby the name of each disorder in figure five of the IUIS classification was searched individually, and together with the terms “alloHSCT” and “transplantation.” We searched for case reports and case series on each disorder, focusing on the question of treatability of the disease by alloHSCT.

## Results

### Group 1: Syndrome-Associated Neutropenia

This group of diseases is dominated by conditions defined by bone marrow failure (BMF), predisposition to myelodysplastic syndrome (MDS) and acute myeloid leukemia (AML), as well as additional non-hematological manifestations such as neurodevelopmental delay (Table 1). Disease control can be achieved for some patients with G-CSF in combination with antibiotic prophylaxis (40, 41). However, the refractoriness of the disease and the predisposition to myeloid malignancies raise the question of if and when curative treatment by alloHSCT is indicated. A lack of evidence regarding long-term outcomes both with and without transplantation, the lack of genotype-phenotype correlation, and the persistence of neurodevelopmental disorders, make recommendations difficult for many of these diseases (Table 1). In some cases only case reports are available, such as in the case of successful transplant of Glycogen Storage Disease type 1b using reduced intensity conditioning (18, 42). On the contrary, more data is available on Schwachman Diamond Syndrome and deficiency of VPS45 protein, and is presented here (43).

**Table 1:** Syndrome-associated neutropenia.

**Disease**	**Gene chromosome inheritance**	**Clinical features**	**G-CSF responsive**	**Risk of progression to MDS/AML**	**Evidence of successful HSCT**	**References**
Schwachman Diamond Syndrome (SDS)	*SBDS* 7q11.21 AR	Bone marrow failure Exocrine pancreas dysfunction Malabsorption Skeletal abnormalities Neurocognitive deficit Recurrent infections (9)	Yes	Yes 15–30% progress to MDS	Yes (see text)	(10–14)
G6PC3 deficiency	*G6PC3* 17q21 AR	SCN Intermittent thrombocytopenia Congential heart disease Urogenital anomalies Dysmorphism Growth and developmental delay Gastrointestinal disease (Crohn's Disease, chronic diarrhea with steatorrhea)	Yes	Yes	Yes	(15–17)
Glycogen storage disease type 1b	*G6PT1* 11q23 AR	Neutropenia Hypoglycemia Recurrent infections Inflammatory bowel disease Liver diseases and hepatosplenomegaly Hypertriglyceridemia	Yes	Yes	Yes	(18, 19)
Cohen syndrome	*VPS13B* (*also called COH1*) 8q22.2 AR	Decreased fetal activity and low birth weight Neutropenia/SCN Obesity (truncal with normal BMI) Hypotonia Dysmorphisms, dental anomalies, poor vision, and limb abnormalities Intellectual disability (severe in 22% of cases)	Yes	None reported	No	(20)
Barth syndrome	*TAZ* Xq28 X-linked	Neutropenia (fluctuating) with occasional monocytosis Dilated cardiomyopathy and rhythm abnormalities Skeletal myopathy Growth delay Developmental delay Hypoglycemia Early death	Yes	None reported	No	(21–23)
Clericuzio syndrome (Poikiloderma with neutropenia)	*USB1* 16q21 AR	Inflammatory eczematous rash (6–12 months) Post-inflammatory Poikiloderma (>2 years) Neutropenia Recurrent sinopulmonary infections and bronchiectasis Nail dystrophy, palmar/plantar hyperkeratosis Reactive airway disease Hypogonadotropic hypogonadism Mid-facial retrusion Calcinosis cutis Non-healing skin ulcers	Yes	Yes	No	(24, 25)
VPS45 deficiency (See text)	VPS45 1q21-22 AR	BMF Neutropenia non-responsive to G-CSF Recurrent, severe bacterial, and fungal infections Extramedullary hematopoiesis with hepatosplenomegaly Nephromegaly	No	Unknown	Yes (see text)	(26–30)
P14/LAMTOR2	*LAMTOR2/MAPBPIP* 3′ UTR p14 AR	SCN Partial albinism B-cell deficiency CD8 deficiency Coarse facial features	Yes	None reported	No	(31)
JAGN1	JAGN1 3p25.3 AR	SCN with increased apoptosis of neutrophils (variable) Recurrent infections Bone, dental, pancreatic insufficiency Failure to thrive Developmental delay	Variable	Yes	Yes (conditioning regimen not specified in literature)	(32, 33)
3-methylglutaconic acid	CLBP 11q13.4 AR	SCN Recurrent infections Progressive brain atrophy with intellectual disability Movement disorder Cataracts Movement disorder	Yes	Yes	No	(34)
SMARCD2	SMARCD2 17q23.3 AR	Neutropenia Delayed separation of the umbilical cord Recurrent infection Chronic diarrhea Developmental delay Dysmorphic features	No	Yes	No	(35)
WDR1	*WDR1* AR 4p16.1	Neutropenia with impaired lymphoid function Mild learning disability Aphthous stomatitis and skin ulcers Pneumonia Gout Pancreatitis Glioblastoma Dysmorphic features in some patients	Unclear	Yes	No	(36–38)
HYOU	*HYOU1* AR 11q23.3	Neutropenia Recurrent oral herpes infection Hypoglycemia Autoimmunity	Yes	None reported	No	(39)

#### Schwachman Diamond Syndrome (SDS)

Schwachman Diamond Syndrome is caused by mutations in the *SBDS* gene at chromosome 7q11.21. Mutations in this gene lead to impaired RNA metabolism and ribosomal function, with clinical features as described in Table 1 (10). *One* third of patients develop major hematological complications (9), and alloHSCT is indicated in cases of bone marrow failure (with subsequent transfusion-dependent anemia, bleeding and severe, recurrent infections), MDS, or AML (11, 12, 44, 45). Numerous studies have shown that alloHSCT is able to correct hematological abnormalities in patients with SDS, however most studies are limited by small sample numbers and/or short follow-up time. A 2005 study by Cesaro et al. (44) reviewed 26 patients with SDS who underwent alloHSCT and demonstrated overall survival of 64.5% at 1 year follow-up. Fifty-six percent of patients who were transplanted following the development of MDS/AML died post-transplant, compared to 19% who were transplanted for BMF, although the difference was not statistically significant. Patients transplanted with total body irradiation (TBI) appeared to do worse than those transplanted with busulfan and fludarabine conditioning, with 67 vs. 20% mortality (*P* = 0.03) (44). In 2002 Hsu reviewed 15 cases of SDS alloHSCT patients and found that transplantation following the development of MDS/AML was associated with a worse outcome; overall survival in this series was 40% (12).

A 2005 review of French registry data found 10 patients who had been transplanted for SDS, five because of bone marrow failure (BMF) and five following development of MDS/AML. They found a 5 years event-free survival of 60% with two patients failing to engraft, one dying 17 months post-HSCT from a respiratory illness, and one death from relapsed MDS. This study noted that patients transplanted prior to the development of malignancy had improved transplant outcomes, and that patients transplanted for BMF demonstrated sustained engraftment with myeloablative regimens based on busulfan and cyclophosphamide. The authors felt that more intense conditioning for BMF-SDS is not warranted, but reduced intensity conditioning for patients with MDS/AML would be insufficient (45). In contrast, Burroughs et al. document three SDS patients transplanted from matched unrelated donors (MUD) with treosulfan and fludarabine conditioning regimens and no serotherapy; these patients underwent transplantation for BMF and achieved sustained engraftment with acute graft vs. host disease (GvHD) developing in two of the three (13).

Thus, for patients with SDS, alloHSCT can correct the hematopoietic aspects of the disease with improved outcomes if transplantation is undertaken prior to development of MDS/AML. Regular monitoring for cytogenetic abnormalities is thus recommended, although some appear to be indolent or transient and are not an automatic indication for HSCT [e.g., i7(q10) and del(20q)]. Larger studies are required before recommendations can be made regarding ideal conditioning regimens (treosulfan or busulfan) and need for serotherapy (particularly when using MUDs) (12, 13, 44, 45).

#### Deficiency of VPS45 Protein

Deficiency of VPS45 protein leads to impaired trafficking of endosomes and lysosomes, with impaired degranulation, release of inflammatory mediators, and neutrophil migration. Patients present very early in life (before 1 year of age) with severe, recurrent, deep-seated bacterial and fungal infections, and a severe neutropenia unresponsive to G-CSF therapy. Bone marrow biopsy classically demonstrates primary myelofibrosis with a dry tap (26–29). HSCT is indicated for severe neutropenia unresponsive to G-CSF and for recurrent, severe infections (27, 30). alloHSCT should be undertaken as early as possible with a myeloablative regimen including busulfan (27). A total of nine transplants have been performed for children with biallelic mutations in the *VPS45* gene, with three deaths and six patients surviving. Surviving patients were transfusion-independent with resolution of the extramedullary hematopoiesis if full donor chimerism was achieved (27).

### Group 2: Neutropenia Without Syndromic Disease

This group includes congenital neutropenia caused by mutations in *ELANE, GFI1, HAX1, WAS* (X-linked neutropenia), *CSF3R*, and *SRP54* genes (Table 2) (40, 43, 46). Several reports on successful alloHSCT are available for both reduced intensity conditioning (RIC), and myeloablative conditioning (MAC) transplantation for this group of diseases (Table 2) and patients younger than 10 years of age appear to have favorable outcomes (7).

**Table 2:** Neutropenia without syndromic disease.

**Disease**	**Gene chromosome inheritance**	**Clinical features**	**G-SCF responsive**	**Risk of progression to MDS/AML**	**Evidence of successful HSCT**	**References**
Severe congential neutropenia 1 (SCN1)	*ELANE* 19p13.3 AD	Neutropenia Recurrent bacterial skin infections Abscess formation Gingivitis Failure to thrive Can also cause cyclic neutropenia	Yes	No	Yes (indicated if high doses of GCSF needed) (see text)	(43)
Severe congential neutropenia 2 (SCN2)	GFI1 1p22.1 AD	Recurrent bacterial skin infections Abscess formation Gingivitis Failure to thrive Mild lymphopenia	Yes	None reported	Not reported	(40, 46, 47)
Severe congenital neutropenia 3 (SCN3)	HAX1 1p22.1 AR	Neutropenia Recurrent bacterial skin infections Gingivitis Failure to thrive Abscess formation Neurological impairment	Yes	Yes	Yes	(43, 48–50)
X-linked neutropenia	*WAS* GOF Xp11.23 X-linked	Neutropenia Bacterial infections Lymphopenia and monocytopenia Autoimmune enteropathy	Yes	Possible (myelodysplasia noted in some patients)	Not reported	(43, 51–53)
G-SCF receptor deficiency	*CSF3R* 1p34.3 AR/acquired in cases of SCN	Cases of acquired somatic mutation in AML/MDS	No	Yes	Annual mutational screening of CSF3R and consideration of HSCT if a mutation was detected.	(43)
Neutropenia with combined immune deficiency	*MKL1* 22q13.1-q13.2 AR	Severe bacterial and fungal infections BCG-related disease Abscess formation Mild thrombocytopenia Failure to thrive	Not reported	Not reported	Not reported	(54)

Severe infections are seen in *ELANE* and *HAX1* mutations, thus alloHSCT is indicated in these patients, particularly if they require high-dose G-CSF to maintain their neutrophil count, or progress to MDS/AML (it has been found that patients who require more than 8 mcg/kg/day of G-CSF to maintain a neutrophil count above 0.5 × 10^9^/L have an increased risk of sepsis and MDS/AML) (43, 46, 55). With regard to *HAX1*, patients with mutations in exon 2 encoding isoform A develop isolated congenital neutropenia, whereas other mutations encoding both isoform A and B cause hematological *and* neurological manifestations (neurological delay, epilepsy). Thus, the degree of neurological impairment should be taken into account when considering alloHSCT (48, 49). Patients with *GFI1* and *WAS* gain of function (GOF) mutations are very rare and seem to present with mild to moderate neutropenia, and there are no reports on alloHSCT in these patients (40, 47). *WAS* GOF (X-linked neutropenia) is a distinct clinical entity from Wiskott Aldrich Syndrome (caused by *WAS* loss of function) with male patients exhibiting variable neutropenia, recurrent infections, and lymphopenia, with normal platelet counts and no eczema. Transient myelodysplastic changes have been seen in the bone marrow of some patients, resolving in most cases but progressing to AML at an elderly age in one case (51–53). In contrast, patients with acquired mutations in *CSF3R* present very often with MDS/AML, therefore yearly screening for receptor mutations is indicated, as is pre-emptive alloHSCT (Table 2) (43).

### Group 3: Phagocyte Function With Syndromic Disease

This group of disease is characterized by defective neutrophil function with normal or elevated neutrophil numbers that is part of a broader syndrome (Table 3). For patients with Papillon-Lefevre, localized juvenile periodontitis, and ß-ACTIN-deficiency there is no need and no evidence for alloHSCT. Patients suffering from leucocyte adhesion deficiencies I and III (LAD) can be cured by alloHSCT, however patients with LADII are not candidates for transplantation. For patients with LADI and III, it appears preferable to transplant early, prior to the development of life-threatening infections. Both RIC and MAC regimens have been successful, although it appears myeloablative reduced toxicity regimens (fludaribine-based, combined with treosulfan, or targeted busulfan dosing) are preferable, with RIC regimens more suitable for sicker patients with reduced Lansky score (61–65). However, a recent study of the EBMT/IEWP on 83 transplanted LAD patients shows no significant benefit in patients receiving MAC vs. non-myeloablation. Furthermore, regardless of the conditioning regimen, a relatively high frequency of severe inflammatory complications (graft rejection and severe aGVHD) during alloHSCT was observed. This cohort included a few LAD III patients, and their outcome was not significantly different from that of the LAD I patients. Early transplantation using anti-inflammtory treatment pre-alloHSCT and the additional use of thiotepa in the conditioning protocol might be beneficial [unpublished data of author SB, (66)]. Following on from successful gene therapy trials for other primary immune deficiencies, a gene therapy phase I/II trial for LAD-1 has been announced for the end of 2019 whereby autologous CD34+ stem cells of patients with LAD1 will be transduced using a lentiviral vector; stem cells successfully transduced will be transplanted following conditioning with a low dose of busulfan (Rocket Pharma, USA; https://clinicaltrials.gov/ct2/show/NCT03812263, https://clinicaltrials.gov/ct2/show/NCT03825783) (67).

**Table 3:** Phagocyte dysfunction with syndromic disease.

**Disease**	**Gene chromosome inheritance**	**Clinical features**	**G-SCF responsive**	**Risk of progression to MDS/AML**	**Evidence of successful HSCT**	**References**
Cystic fibrosis transmembrane conductance regulator (CFTR)—dependent Leukocyte adhesion deficiency type IV (LAD IV)	*CFTR* 7q31.2 AR	Clinical features of cystic fibrosis	No	No	No	(56, 57)
Papillon-Lefevre	*CTSC* 11q14.2 AR	Palmoplantar keratoderma Periodontitis Premature loss of dentition Liver abscesses Pneumonia	Unknown	No	No	(58)
Localized juvenile periodontis	*FPR-1* 11q14.1 AR	Palmoplantar keratoderma Periodontitis Premature loss of dentition Liver abcess	Yes	No	No	(59)
ß-ACTIN	*ACTB* 7p22.1 AD	Developmental delay Recurrent infections Photosensitivity Thrombocytopenia Stomatitis	Unknown	Unkown	No	(60)
Leukocyte adhesion deficiency type I (LAD I)	*ITGB* 12q13.13 AR	Severe leucocytosis Severe recurrent bacterial infections impaired pus formation Delayed wound healing Delayed umbilical cord detachment	No	No	Yes Complicated by aGVHD Indicated in young patients with MSD, RIC/MAC both used Additional Thiotepa beneficial	(61–63)
Leukocyte adhesion deficiency type II (LAD II) Also known as CDG2C	*SLC35C1* 11p11.2 AR	Severe leucocytosis Recurrent bacterial infection Moderate to severe psychomotor retardation Mild to severe dysmorphism Impaired neutrophil motility Bombay blood group	No	No	No	(61–63)
Leukocyte adhesion deficiency type III (LAD III)	*FERMT3* 11q13.1 AR	Severe leucocytosis Recurrent bacterial infection Bleeding tendency	No	No	Yes Complicated by aGVHD, good in young patients with MSD, RIC/MAC both used, additional thiotepa indicated	(61–64)

### Group 4: Phagocyte Dysfunction Without Syndromic Disease

Excluding CGD, the only disease in this group with evidence of successful bone marrow transplantation is MonoMAC (monocytopenia and mycobacterial infections syndrome) (Table 4). This disease is characterized by profoundly decreased or absent monocytes, B lymphocytes, natural killer (NK) cells, and circulating and tissue dendritic cells (DCs), with little, or no effect on T-lymphocyte numbers. Patients are susceptible to mycobacterial, viral and opportunistic fungal infections. Bone marrow dysfunction is prominent and variable with progressive aplastic and dysplastic changes. Stem cell transplantation is curative and can be performed pre-emptively in cases where a matched donor is available (68). Anti-inflammatory pre-treatment and proper infection control might result in lower transplant related mortality in these patients.

**Table 4:** Phagocyte dysfunction without syndromic disease (excluding CGD).

**Disease**	**Gene chromosome inheritance**	**Clinical features**	**G-SCF responsive**	**Risk of progression to MDS/AML**	**Evidence of successful HSCT**	**References**
MonoMac	*GATA2* 3q21.3 AD	Infections Cytopenia (including monocytopenia) Lymphedema Pulmonary alveolar proteinosis Deafness Predisposition to mycobacteria	No	Yes	Yes	(43, 68)
Specific granule deficiency	*CEBPE* 14q11.2 AR *SMARCD2* *SMARCD2* 17q23.3AR	Recurrent infections, neutropenia, Dysmorphic features, developmental delay	No	Yes	No	(15–17, 35, 69, 70)
Neutrophil immune deficiency Syndrome	*RAC2* 22q13.1 AD	Severe bacterial infection Poor wound healing Absence of pus	Unclear	No	No	(70, 71)
G6PD deficiency class I	*G6PD* Xq28 X-linked	Severe hemolytic anemia in response to specific medications and fava beans Chronic anemia	No	No	No	(72)

## Discussion

The list of neutrophil disorders is varied and expanding rapidly with the increasing availability of next generation gene sequencing. As our ability to establish a genetic diagnosis is improved, the challenge of linking genotype to phenotype arises, a challenge that is made difficult by the small numbers of patients diagnosed with each individual disease. There is much we still do not understand about the pathomechanism of these diseases, and this makes prognostication and treatment decisions difficult. Serious infectious complications and neutropenia are life threatening but can be treated with anti-microbial therapy and G-CSF, respectively, however inflammatory complications of these diseases are likely underappreciated and undertreated (5, 6). Furthermore, the decision to undertake alloHSCT, with all its inheritent risks and potential complications, is made difficult by the lack of published data for most diseases.

In 2015, the EBMT and SCETIDE released the findings of the largest retrospective cohort of patients with severe congenital neutropenia to undergo alloHSCT. The 136 patients in that study demonstrated an overall 3 years survival of 82%, with transplant related mortality at 17%. It concluded that transplantation should be considered in patients with severe infections or unresponsiveness to G-CSF or requiring high doses of G-CSF (over 8 mcg/kg/day to maintain an absolute neutrophil count over 0.5 × 10^9^/L). Both MAC and RIC conditioning was effective and transplant outcomes were improved if patients were transplanted before 10 years of age and before the development of MDS/AML (7). Despite the wide range of disease we have covered in this review, those findings appear to hold true, although in diseases where evidence is lacking family choice and center-specific expertise must also be taken into account.

In diseases where the need to transplant has been established (e.g., deficiency of VPS45 protein, LAD, SDS), there is little to guide decision making as to the method of transplantation. The need to achieve stable myeloid engraftment, often into an impaired bone marrow environment, may well call for the use of toxicity reduced MAC over the RIC regimens that have become established practice for PIDs characterized by loss-of-function lymphoid deficiencies (e.g., severe combined immunodeficiency, or SCID) (27). Again, evidence is lacking regarding side effects, long-term outcomes, and the degree of chimerism required to maintain cure. Secondary graft failure or graft insufficiency in the case of mixed chimerism with a reappearance of post-transplant neutropenia has been reported in the literature and observed by the authors in some of the quantitative phagocytic disorders (73–75).

We recommend that all physicians treating patients with neutrophil disorders submit their data to the EBMT and SCETIDE, as it is only through the ordered collection of data that clinical experience can be gathered, studied and shared. It is our hope that in the coming years many disease mechanisms will be uncovered and long-term treatment data accumulated, which will be of great benefit to physicians, patients, and their families.

## Author Contributions

SB, BS, and PS designed the study, collected data, performed review, wrote the manuscript.

### Conflict of Interest

The authors declare that the research was conducted in the absence of any commercial or financial relationships that could be construed as a potential conflict of interest. The handling editor, AG, declared a collaboration with one of the authors, SB.

## Abbreviations

AD: Autosomal Dominant
aGVHD: Acute Graft vs. Host Disease
alloHSCT: Allogeneic Hematopoietic Stem Cell Transplant
AML: Acute Myeloid Leukemia
AR: Autosomal Recessive
BMF: Bone Marrow Failure
CGD: Chronic Granulomatous Disease
cGVHD: Chronic Graft vs. Host Disease
CHD: Congenital Heart Disease
EBMT: European Society for Bone Marrow Transplantation
ESID: European Society for Immunodeficiencies
G-CSF: Granulocyte Colony Stimulating Factor
GOF: Gain of Function
GVHD: Graft vs. Host Disease
HSCT: Hematopoietic Stem Cell Transplantation
IEWP: Inborn Errors Working Party
IUIS: International Union of Immunological Studies
LAD: Leukocyte Adhesion Deficiency
MAC: Myeloablative Conditioning
MDS: Myelodysplastic Syndrome
MUD: Matched Unrelated Donor
PID: Primary Immune Deficiency
RIC: Reduced Intensity Conditioning
SAA: Severe Aplastic Anemia
SCETIDE: Stem Cell Transplant for Immunodeficiencies in Europe
SCN: Severe Congenital Neutropenia
SDS: Schwachman Diamond Syndrome
TBI: Total Body Irradiation.
